# Myofiber branching rather than myofiber hyperplasia contributes to muscle hypertrophy in *mdx* mice

**DOI:** 10.1186/2044-5040-4-10

**Published:** 2014-05-23

**Authors:** Rachel M Faber, John K Hall, Jeffrey S Chamberlain, Glen B Banks

**Affiliations:** 1 Department of Neurology, University of Washington, Seattle, WA 98195-7720, USA; 2 Department of Biochemistry, University of Washington, Seattle, WA 98195-7720, USA; 3 Department of Medicine, University of Washington, Seattle, WA 98195-7720, USA

**Keywords:** Duchenne muscular dystrophy, Skeletal muscle, Hypertrophy, Fiber branching, Innervation, Neuromuscular synapse, Inflammation

## Abstract

**Background:**

Muscle hypertrophy in the *mdx* mouse model of Duchenne muscular dystrophy (DMD) can partially compensate for the loss of dystrophin by maintaining peak force production. Histopathology examination of the hypertrophic muscles suggests the hypertrophy primarily results from the addition of myofibers, and is accompanied by motor axon branching. However, it is unclear whether an increased number of innervated myofibers (myofiber hyperplasia) contribute to muscle hypertrophy in the *mdx* mice.

**Methods:**

To better understand the cellular mechanisms of muscle hypertrophy in *mdx* mice, we directly compared the temporal progression of the dystrophic pathology in the extensor digitorum longus (EDL) muscle to myofiber number, myofiber branching, and innervation, from 3 to 20 weeks of age.

**Results:**

We found that a 28% increase in the number of fibers in transverse sections of muscle correlated with a 31% increase in myofiber branching. Notably, the largest increases in myofiber number and myofiber branching occurred after 12 weeks of age when the proportion of myofibers with central nuclei had stabilized and the *mdx* mouse had reached maturity. The dystrophic pathology coincided with profound changes to innervation of the muscles that included temporary denervation of necrotic fibers, fragmentation of synapses, and ultra-terminal axon sprouting. However, there was little evidence of synapse formation in the *mdx* mice from 3 to 20 weeks of age. Only 4.4% of neuromuscular junctions extended ultra-terminal synapses, which failed to mature, and the total number of neuromuscular junctions remained constant.

**Conclusions:**

Muscle hypertrophy in *mdx* mice results from myofiber branching rather than myofiber hyperplasia.

## Background

Duchenne muscular dystrophy (DMD) is the most common dystrophy affecting approximately 1:4,000 boys, and is caused by mutations in the dystrophin gene [1,2]. A characteristic feature of DMD patients is the transitory hypertrophy of certain muscle groups, such as the gastrocnemius muscle. Direct correlations between computer tomography and histology of muscle biopsies demonstrate that an increase in myofiber number contributes to a transitory period of true hypertrophy in some DMD patients [3-5]. Ultimately, the failure of the muscle stem cells to continually regenerate the necrotic fibers leads, in part, to the replacement of muscle with adipose and connective tissue [6-9]. Muscle hypertrophy is found in most muscles in the *mdx* mouse model of DMD between 10 and 40 weeks of age and functionally compensates, in part, for the lack of dystrophin [5,10-13]. While the area of individual myofibers in *mdx* mice is highly variable, the average area is unchanged when compared to wild-type myofibers [14]. Similar to the DMD patients, estimations of myofiber number in transverse sections of *mdx* muscles are increased when compared to wild-type myofibers [12]. The endogenous mechanisms for increasing the number of myofibers is of considerable interest for muscle replacement strategies to treat muscular dystrophy and sarcopenia. However, it is unclear whether the additional muscle results from myofiber branching and/or from the formation of new, innervated myofibers (myofiber hyperplasia).

Several considerations suggest that the increase in myofiber number in *mdx* mice could result from myofiber hyperplasia. Satellite cells associated with myofibers can begin proliferating and differentiate into new myotubes in culture [15]. Furthermore, proliferative expansion of an activated satellite cell *in vivo* can contribute to the regeneration of clusters of adjacent fibers [16-18]. Muscle necrosis in *mdx* mice can initiate the expression of growth-associated protein 43 (GAP43), a marker of axonal branching, in motor neurons [19]. While satellite cells are evenly distributed along a myofiber, a satellite cell resides in close proximity to the neuromuscular junction [20]. Innervation of regenerating fibers may be beneficial as direct stimulation of muscle can improve the engraftment of satellite cells [21]. Finally, myofibers can generate force through myomuscular junctions without having to extend from tendon to tendon [22]. Here, we directly compared the temporal progression of dystrophic pathology in *mdx*^*4cv*^ mice to innervation, myofiber number, and myofiber branching. Our results demonstrate that the increase in myofiber number in transverse sections of *mdx* EDL muscles results from myofiber branching rather than myofiber hyperplasia.

## Methods

### Mice

We generated the *mdx*^*4cv*^: Thy1-CFP mice by breeding *mdx*^*4cv*^ C57Bl/6 females with C57Bl/6 mice homozygous for the Thy1-CFP transgene [23]. We utilized the male offspring, which were all *mdx*^*4cv*^ and expressed the CFP transgene under the control of the Thy1 promoter. For myofiber branching experiments we utilized *mdx*^*4cv*^ mice expressing the DsRed transgene in every cell. Mice were genotyped as previously described [24]. All experiments were performed in accordance with the guidelines approved by the Institutional Animal Care and Use Committee of the University of Washington.

### Wholemount immunostaining

Wholemount immunostaining was performed as previously described [25]. Briefly, the *mdx*^*4cv*^: Thy1-CFP mice were examined at 3 weeks (n = 4), 4 weeks (n = 4), 12 weeks (n = 6), and 20 weeks of age (n = 4). The mice were anesthetized with 2,2,2-tribromoethanol and perfused with 2% paraformaldehyde (PFA) in 1× phosphate buffered saline (PBS) by cardiac infusion. Cervical dislocation of the mouse was immediately followed by dissection of the lower limb such that the extensor digitorum longus (EDL) was the only muscle still attached to the bone and was incubated for 2 hours in 2% PFA at 4°C. The EDL muscles were excised from the bone and the distal tendons were isolated. The fascicular outlines of four muscle groups can be followed from tendon to tendon [26]. We stripped each of the four muscle groups that comprise the EDL using the distal tendons and retained the third group that normally inserts on the distal phalanx of the third digit of the foot. The third compartment of the EDL was incubated in 0.1 M glycine (in 1× PBS) for 1 hour and in blocking solution (1% BSA, 0.05% triton X-100, 1× PBS) overnight at 4°C. The muscles were then incubated in α-bungaratoxin conjugated to tetramethylrhodamine (TRITC; 1:800; Life Technologies) and GAP43 conjugated to DyLight 650 (1:800; Novus Biologicals) at room temperature on a rotating platform for 1 hour. For analyses of immune cells the muscles were incubated in Alexa-647 conjugated CD4, FITC-conjugated interferon-γ, Alexa488-conjugated CD8a, or FITC conjugated Mac-1 (1:50; BD Pharmingen). The muscles were then washed 3 × 10 min in 1× PBS and loaded onto superfrost^+^ slides with ProLong Gold antifade mounting medium containing 4′,6-diamidino-2-phenylindole (DAPI; Life Technologies). A composite image of the entire third muscle compartment was visualized and imaged using a Leica SP5 confocal microscope.

### Quantitation of synapses

We quantitated the total number of synapses in the wholemount images using the count tool in Adobe Photoshop. Ultra-terminal branches were defined as motor axons that extended from the synapse. Those that terminated on an AChR cluster were labeled synaptic and those that did not terminate on an AChR cluster were labeled non-synaptic. The proportion of ultra-terminal synapses was calculated by dividing the total number of ultra-terminal synapses by the total number of neuromuscular junctions.

### Quantitation of myofibers

Once the innervation of the third compartment of the EDL had been imaged the slide was incubated overnight in 2% paraformaldehyde in 1× PBS on a rotating table at room temperature to allow the coverslip to release from the slide without affecting the integrity of the muscle. The muscle was then placed in 20% sucrose for 2 hours at 4°C and frozen in OCT in 2-methylbutane in liquid N_2_. Adjacent 10 μm sections were stained with hematoxilyn and eosin or wheat germ agglutinin (WGA) conjugated to fluorescein isothyocyanate (FITC; 1:100; Vector Laboratories). The sections stained with WGA were washed 3× 10 minutes in 1× PBS, mounted with ProLong Gold antifade mounting medium (Life Technologies). The sections were imaged using a Nikon Eclipse E1000 fluorescent microscope. The total number of myofibers in the third compartment of the EDL was quantitated using the slides stained with WGA using the count tool in Adobe photoshop. The myofiber areas were measured using the FIJI computer software (NIH). We measured the areas of 167 myofibers from n = 4 wild-type mice and 265 myofibers from n = 4 *mdx* mice.

### Analyses of branched fibers

The entire EDL muscles from *mdx*^*4cv*^*:* DsRed mice aged 3 weeks, 4 weeks, 12 weeks, and 20 weeks were immediately digested in 6,000 Units of collagenase II (Worthington; NJ) for 60 minutes at 37°C with gentle agitation every 15 minutes. The collagenase II solution was exchanged with 1× PBS containing Alexa488-conjugated αBTX (1:800; Life Technologies) and incubated on a rotating table for 1 hour at room temperature. The muscles were washed three times in 1× PBS. The DsRed was expressed in every fiber, but quickly disappeared upon necrosis. The healthy DsRed fluorescing fibers were then carefully teased on a superfrost^+^ slide in ProLong Gold mounting medium containing DAPI. The fibers were quantitated and imaged using a Leica SP5 confocal microscope to gain sufficient resolution in the Z-direction as well as the X/Y planes. We quantitated 289 fibers at 3 weeks of age (n = 5), 256 fibers at 4 weeks (n = 3), 365 fibers at 12 weeks (n = 6), and 327 fibers at 20 weeks (n = 4). A myofiber with multiple branches was quantitated as a single branched myofiber. We also compared the myofiber diameters of unbranched (61 myofibers) and the combined diameters of branched myofibers (42 myofibers) in teased muscle preparations from n = 4, 5-month-old *mdx*^*4cv*^: DsRed EDL using FIJI.

### Statistics

Comparisons of muscle mass and myofiber areas were compared using a Students *t*-test. Myofiber number, ultra-terminal axon branches, and myofiber branching were analyzed using a one-way ANOVA with a Tukey post-test. Direct comparisons of synapse number with myofiber number were made using a two-way ANOVA with a Tukey post-test. All data analyses were performed using the PRISM software.

## Results

### Temporal progression of dystrophic pathology

To better understand the cellular mechanisms of muscle hypertrophy in *mdx* mice we directly compared the number of myofibers to the number of neuromuscular junctions. To visualize innervation of skeletal muscles we generated *mdx*^*4cv*^: Thy1-CFP mice that express CFP in the motoneurons. We chose to examine the extensor digitorum longus (EDL) muscle as the anatomy of the EDL is well described [26]. The EDL has a single proximal tendon that attaches the lateral epicondyle of the distal femur to the distal phalanx of the foot through four distal tendons. The four muscle fascicles extend from tendon to tendon and can be easily and consistently separated. We chose the third muscle group because it is flat, small, and amenable to future muscle stem cell treatments.

**Figure 1:**
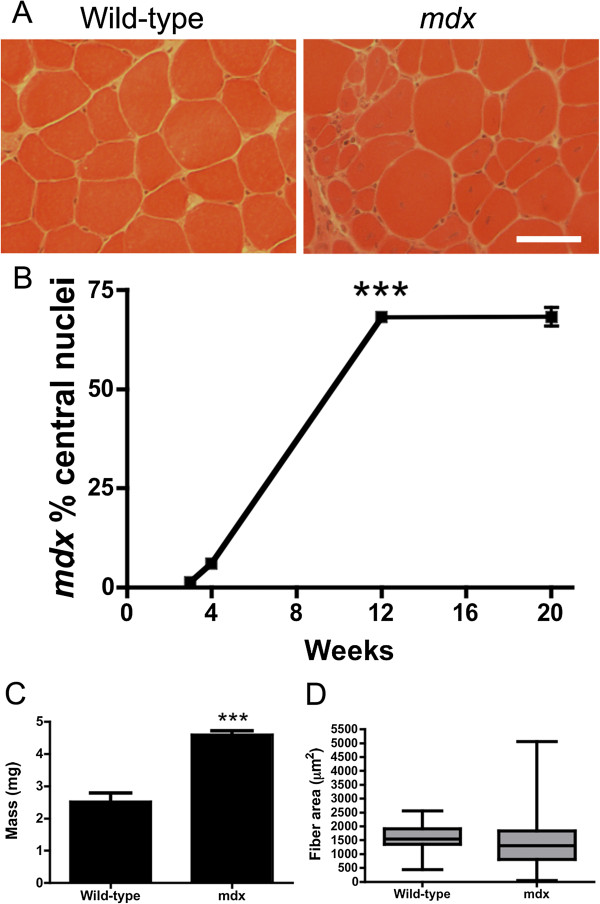
**Progression of dystrophic histopathology in the third compartment of the extensor digitorum longus (EDL) muscle. (A)** Hematoxylin and eosin stained frozen sections from 3-month-old wild-type and *mdx*^*4cv*^ muscle. Note the central nuclei in the *mdx*^*4cv*^ myofibers. Scale bar = 50 μm. **(B)** Proportion of central nuclei (+/- S.D.) in the third EDL compartment from 3 weeks to 20 weeks of age (n = 4-6). Note the apparent stabilization of dystrophic histopathology from 12 to 20 weeks of age. ***P <0.001 at 12 weeks compared to 4 weeks of age. At some time points, the standard deviation is too small to see. **(C)** The mass of the third compartment of the EDL at 20 weeks of age. *P* <0.001 compared to wild-type. **(D)** Box-whiskers plot of median +/- 75% of myofiber areas in wild-type and *mdx* mice at 20 weeks of age.

Transverse sections of the third compartment of the wild-type: Thy1-CFP EDL revealed that less than 1% of the myofibers had centrally located nuclei, a marker of skeletal muscle regeneration (Figure 1A). Centrally located nuclei in the *mdx*^*4cv*^: Thy1-CFP EDL were evident by 4 weeks of age (6% of muscles), while their numbers had peaked by 12 weeks (68%; *P* <0.001), and had stabilized until at least 20 weeks of age (68%; Figure 1). Thus, we chose 3 weeks (onset of necrosis), 4 weeks (evidence of regeneration), 12 weeks (peak of central nucleation), and 20 weeks (stabilization of dystrophic pathology) for the time points for this study. The third compartment of the EDL mass was increased by 82% in *mdx* compared to wild-type at 20 weeks of age (Figure 1C). While the myofiber areas were highly variable in the *mdx* mice at 20 weeks of age, the average areas remained unchanged (*P* = 0.71). Thus, the dystrophic pathology in the third compartment of the EDL led to significant hypertrophy in *mdx* mice, which did not result from changes in the average myofiber area.

### Synaptic remodeling in mdx^4cv^: Thy1-CFP mice

**Figure 2:**
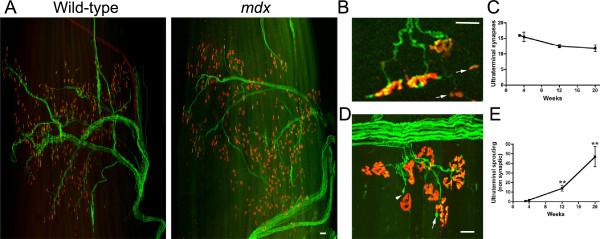
**Innervation in the third EDL compartment. (A)** The transgenic cyan fluorescence protein expression in neurons is pseudo-colored in green to demonstrate the innervation patterns in wild-type and *mdx*^*4cv*^ muscles at 4 weeks of age. Note the profound infiltration of small acetylcholine receptor (AChR) patches in synaptic and non-synaptic regions within the *mdx*^*4cv*^ muscles. Scale bar = 100 μm. **(B)** Shown are examples of ultra-terminal synapses (arrows) in 4-week-old muscle. Note that the ultra-terminal synapses are immature plaques. **(C)** Graph shows the mean +/- S.D. total number of ultra-terminal synapses demonstrating a modest reduction with age (*P* = 0.056; n = 4-6). **(D)** Shown is an example of denervation (arrow head) and non-synaptic ultra-terminal branching (arrow) in 20-week-old muscle. **(E)** The mean +/- S.D. total number of non-synaptic ultra-terminal branches increased with age (n = 4-6). **P <0.01 compared to 4 weeks of age. Scale bar for **B** and **D** = 30 μm.

High-resolution confocal images of wholemount preparations of the third compartment of the EDL revealed that the motor nerve trunk traverses the skeletal myofibers to innervate the postsynaptic acetylcholine receptor (AChR) clusters (Figure 2A). The transgenic expression of CFP does not influence the innervation patterns in mice [23]. The innervation patterns were qualitatively normal when comparing wild-type and *mdx*^*4cv*^: Thy1-CFP muscles at 4 weeks of age (Figure 2A). At 3 to 4 weeks of age we noticed a large number of small extrasynaptic AChR clusters within the *mdx*^*4cv*^: Thy1-CFP muscles (Figure 2A). Detailed analyses of the wholemount preparations revealed that mononuclear inflammatory cells including activated CD4 positive T-helper cells expressing interferon-γ (Additional file 1: Figure S1A), CD8 positive cytotoxic T-lymphocytes (Additional file 1: Figure S1B), or macrophages (Additional file 1: Figure S1C) could express AChR, as previously described [27]. We found no direct association between the immune cells and the neuromuscular junctions.

The generation of new, innervated fibers requires synapse formation in the postnatal mice. Therefore, we performed a detailed examination of the wholemount preparations for evidence of synapse formation in *mdx*^*4cv*^: Thy1-CFP EDL muscles. Neuromuscular junctions form as a plaque, and mature into a donut shape, and ultimately to a pretzel-like profile [28]. The presynaptic motor nerve terminal directly apposed the postsynaptic AChRs in wild-type muscles (Figure 2A) [29]. While the formation and maturation of the primary postsynaptic apparatus of the neuromuscular junction was normal in *mdx* mice (Figure 2A) [30], we found that motor axons could extend beyond the synapse (ultra-terminal axon sprouting) (Figure 2B-E). On average, only 4.4% of the ultra-terminal axon sprouts innervated immature secondary synaptic plaques (Figure 2B). The number of ultra-terminal synapses reduced from approximately 16 per muscle (5.1%) at 3 weeks of age to approximately 12 per muscle (3.9%) at 20 weeks of age in *mdx* mice (*P* = 0.056; one-way ANOVA; Figure 2C). Furthermore, we found no evidence for the maturation of the ultra-terminal synaptic plaques to a donut or pretzel profile in any of the muscles examined. Most ultra-terminal axon sprouts in *mdx*^*4cv*^: Thy1-CFP muscles did not terminate on to AChR clusters (Figure 2D, E). Non-synaptic ultra-terminal axon sprouts significantly increased with age, with approximately one per muscle at 3 weeks of age up to approximately 47 per muscle at 20 weeks of age (Figure 2D, E; *P* <0.01; one-way ANOVA). Thus, ultra-terminal synapse formation was evident in the *mdx* EDL muscles, but they were few in number, decreased with age, and failed to mature.

**Figure 3:**
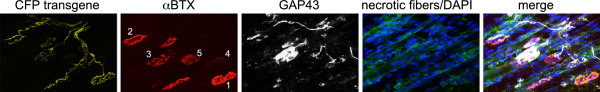
**Skeletal muscle necrosis initiates temporary denervation.** The CFP is pseudo-colored in yellow to allow the visualization of multiple fluorophores. Necrotic IgG positive fibers are shown in green. Note we numbered the synapses in the second panel. Synapse 1: Normal synapse in a non-necrotic fiber (no IgG). Synapse 2: Expression of GAP43 in a pre-necrotic fiber (IgG is next to the fiber). Synapse 3: Onset of necrosis (green fiber without overt cellular infiltrate) leads to the activation of the Schwann cell expressing GAP43 that retracts the presynaptic nerve terminal from the postsynaptic acetylcholine receptors. Synapse 4: Infiltration of cells into the necrotic fibers led to complete retraction of the nerve and loss of the AChR clusters. Synapse 5: Re-innervation of a fragmented postsynaptic synapse in a post-necrotic fiber (note the central nuclei and absence of IgG (green)). Scale bar = 30 μm.

GAP43 is expressed in newly growing motor axons, terminal Schwann cells, immune cells, and parasympathetic nerves in blood vessels [19]. To determine if skeletal muscle necrosis in *mdx*^*4cv*^: Thy1-CFP EDL muscles could initiate motor axon branching and synaptogenesis we examined the synapses at 3 and 4 weeks of age in wholemount preparations. It is well established that synapses transition from the normal pretzel profile to the fragmented grape appearance upon muscle necrosis in *mdx* mice [30,31], but what happens in between to both the pre- and postsynaptic apparatus is unclear. Here, we found that the onset of skeletal muscle necrosis, as determined by Alexa488-IgG positive fibers, led normal pretzel profiled synapses (for example, Figure 3, synapse 1) to express GAP43 in the presynaptic nerve terminals (for example, Figure 3, synapse 2, 3, and 4). The early stage of necrosis, as determined by the localization of IgG on the surface of the muscle without overt cellular infiltrates, was accompanied by the retraction of the presynaptic nerve terminal from the postsynaptic apparatus (for example, Figure 3, synapse 3). The GAP43 in synapse 3 is expressed in the terminal Schwann cell, as previously described [19]. Infiltration of mononuclear cells into the necrotic myofibers led to the loss of the postsynaptic AChR clusters and the presynaptic nerve terminal (for example, Figure 3, synapse 4). Another example of the loss of innervation can be found in Figure 2D (arrow head). The regenerating fibers, as determined by the central nucleation without surface localized IgG, were reinnervated with a fragmented grape-profiled pre- and postsynaptic apparatus (for example, Figure 3, synapse 5). Thus, skeletal muscle necrosis in *mdx*^*4cv*^: Thy1-CFP EDL muscles initiated a temporary denervation of the myofibers rather than directly evoking motor axon branching from the nerve terminals.

### Direct comparison of myofiber number to innervation

**Figure 4:**
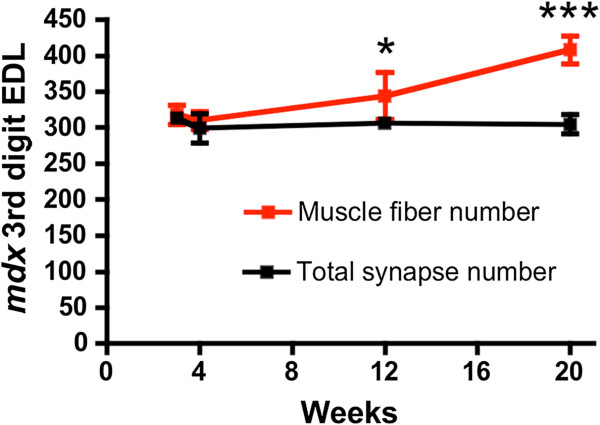
**Direct comparison of myofiber number with synapse number in the third EDL compartment.** Note that myofiber number in transverse sections increases with age while the synapse number remains unchanged (n = 4-6). *P <0.05 and ***P <0.001 fiber number compared to synapse number.

The number of myofibers and the number of neuromuscular junctions in the wild-type EDL remains constant in maturing wild-type mice [32] (Additional file 2: Figure S2). Despite the profound changes in motor axon plasticity in the *mdx*^*4cv*^: Thy1-CFP EDL muscles, we found no significant change in the total number of neuromuscular synapses from 3 weeks to 20 weeks of age (Figure 4; *P* = 0.65 one-way ANOVA). To directly compare the number of synapses to the number of myofibers we reprocessed the same EDL muscle compartment for histological sections and stained the sections with wheat germ agglutinin conjugated to fluorescein isothyocyanate (FITC). We examined sections through the mid-belly of the EDL to coincide with the placement of the synapses. Quantitation of the total number of *mdx*^*4cv*^: Thy1-CFP myofibers revealed a 28% increase from 3 weeks to 20 weeks of age (Figure 4; *P* <0.001; one-way ANOVA). Interestingly, the total number of fibers increased by 19% from 12 weeks to 20 weeks of age when the dystrophic histopathology had seemingly stabilized (Figure 1). Thus, an increase in myofiber number in hypertrophic *mdx* muscles was not accompanied by a change in the total number of neuromuscular junctions.

### Increased myofiber numbers in *mdx*^*4cv*^: Thy1-CFP mice result from myofiber branching

**Figure 5:**
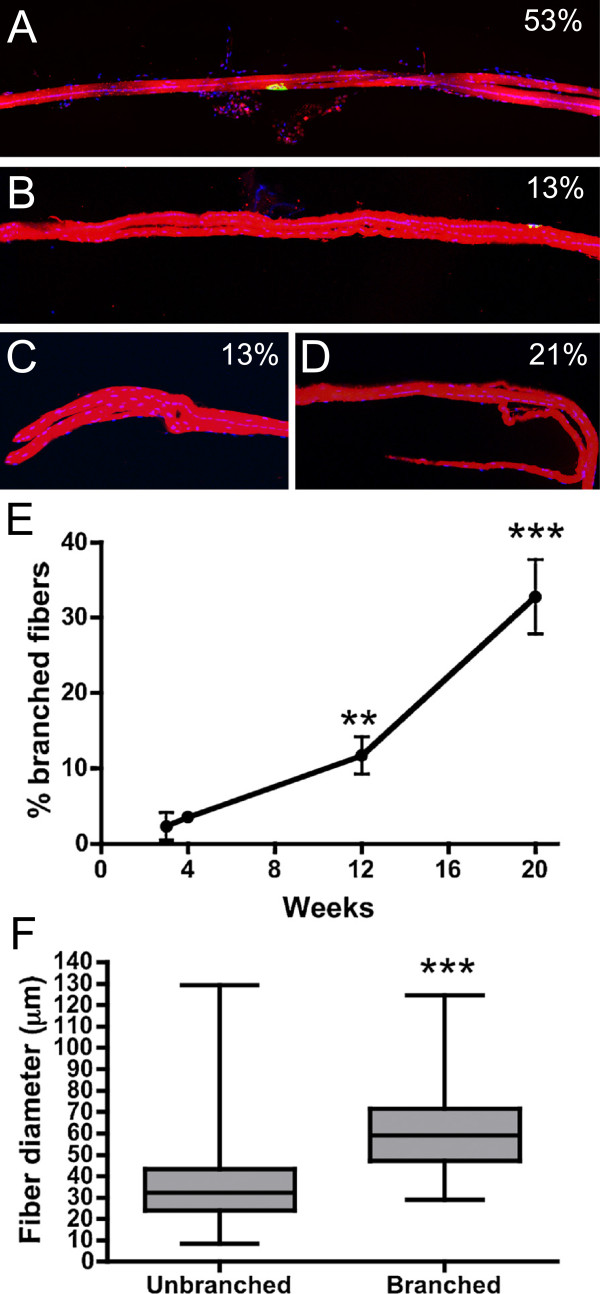
**Myofiber branching in the EDL. (A)** A singularly innervated branched myofiber expressing DsRed, DAPI in blue and AChR cluster in green. Scale bar = 50 μm. **(B)** Branches can also rejoin the initial fiber and/or **(C)** branch at the ends of myofibers. Percentages in the images in **A-C** show the proportion of myofibers with the respective branching phenotype. **(D)** Shown is an example of a myofiber with multiple branch points. Approximately 21% of myofibers with branches had multiple branch points. **(E)** Mean +/- S.D. of fiber branching increases with age. **P <0.01 compared to 4 weeks and ***P <0.001 compared to 12 weeks (n = 4-6). **(F)** Median +/- 75% diameter of unbranched *mdx* EDL myofibers compared to the combined diameter of branched myofibers at 5 months of age. ***P <0.001.

We next examined whether the increase in myofiber number in transverse sections of the third compartment of the *mdx*^*4cv*^: Thy1-CFP EDL muscles correlated with an increase in myofiber branching. Here, we digested the entire *mdx*^*4cv*^: Thy1-CFP EDL muscles with collagenase II and quantitated the proportion of branched fibers (Figure 5). To easily count the muscles we utilized the *mdx*^*4cv*^: Thy1-CFP expressing DsRed. We found three types of myofiber branching including branching from the middle of the myofiber (53% of myofibers with branches; Figure 5A), branches that re-entered the original myofiber (13% of myofibers with branches; Figure 5B), and myofiber branching at the ends of the myofiber (13% of myofibers with branches; Figure 5C). Of the myofibers with branches, 21% had multiple branch points (Figure 5D), with various combinations of the above. We found that the proportion of branched red myofibers increased from 2% at 3 weeks of age to 33% at 20 weeks of age (31% increase; *P* <0.001; one-way ANOVA), which was consistent with the 28% increase in the number of fibers in transverse sections. Interestingly, the proportion of branched fibers increased by 21% between 12 weeks and 20 weeks of age (Figure 5E), when the dystrophic histopathology had stabilized (Figure 1). The combined average diameter of a branched myofiber was increased by 76% when compared to the unbranched *mdx* myofibers at 5 months of age demonstrating that myofiber branching led to myofiber hypertrophy (Figure 5F; *P* <0.001). While we found that 1.4% of the unbranched fibers had multiple synapses (Additional file 3: Figure S3), all of the branched fibers were singularly innervated at all ages examined (Figure 5), as previously described [33]. Thus, the temporal increase in myofiber number in transverse sections of *mdx*^*4cv*^: Thy1-CFP muscles directly correlated with the increased number of branched myofibers.

## Discussion

The transitory muscle hypertrophy in *mdx* mice can lead to a partial functional compensation for the lack of dystrophin [5,10-13]. For example, larger muscles in *mdx* mice generate greater force than in wild-type mice, but they do not develop greater levels of specific force and they continue to display cycles of necrosis and regeneration [13]. Muscle necrosis leads to a potent regenerative response in *mdx* mice that leads to the functional hypertrophy [6-9]. To better understand the cellular mechanisms for muscle hypertrophy in *mdx* mice we directly compared the temporal progression of dystrophic pathology in the third compartment of the EDL in *mdx*^*4cv*^: Thy1-CFP mice to myofiber number, innervation, and myofiber branching. Similar to previous studies we found an increase in the number of myofibers in transverse sections [12]. Despite profound changes to the innervation of *mdx* muscles, there was no change in the total number of neuromuscular junctions. Rather, the increase in myofiber number quantitated in transverse sections correlated with an increase in myofiber branching. Thus, our data suggest that myofiber branching rather than the formation of new, innervated myofibers (myofiber hyperplasia) contributes to the muscle hypertrophy in *mdx* mice.

Fiber branches can form through the generation of a new myofiber segment physically connected to a pre-existing myofiber or by splitting a fiber into two. Consistent with the former possibility, we found that all branched fibers in the *mdx* mice had central nuclei in at least one of the branches (Figure 5). Furthermore, fiber branches in *mdx* mice express developmental myosin [33]. Activated satellite cells could potentially generate the fiber branches by fusing into nascent or pre-existing myofibers in a non-linear pattern [34]. Satellite cells are also required for the increase in number of fibers that is associated with experimental induction of muscle hypertrophy by mechanical overload [35]. Our results confirm previous reports that the most severe dystrophic histopathology occurs during maturation of the mouse from 3 to 12 weeks of age [12,36-38]. Interestingly, the greatest changes in myofiber number and myofiber branching occurred between 12 and 20 weeks of age when cycles of necrosis and regeneration had reached a steady state level. The stabilization of dystrophic histopathology during this later period requires the activation of satellite cells to support myofiber regeneration [12]. Therefore, it is likely that the potent regenerative response in *mdx* muscles leads to hypertrophy through the generation of myofiber branches rather than forming entirely new innervated myofibers.

While skeletal muscle necrosis can lead to overt changes in GAP43 expression in *mdx* muscles [19], it was unclear whether this represented overt changes to innervation of the skeletal muscles because GAP43 can label multiple cell types. Our detailed examination of wholemount preparations demonstrated profound changes to the innervation of *mdx*^*4cv*^: Thy1-CFP muscles. We found that GAP43 was expressed in the motor nerves and terminal Schwann cells during the temporary denervation of the necrotic fibers. The terminal Schwann cell normally envelops the motor nerve terminal when synapses are eliminated during early postnatal development [39]. A similar process in *mdx* mice could potentially function to protect the nerve terminal from the profound inflammatory response within the muscle [40]. Skeletal muscle necrosis did not directly elicit ultra-terminal axon sprouting, which was prevalent at all ages examined. Only a small portion of these axonal extensions formed ultra-terminal synaptic plaques, which never developed into the donut or pretzel profiles of maturing synapses. The non-synaptic ultra-terminal axon sprouting was most prevalent between 12 and 20 weeks of age similar to the time course of myofiber branching. The excitation-contraction coupling is disturbed in branched myofibers [33], and this could potentially impair synaptic transmission leading to the presynaptic axonal extensions. We found that branched myofibers were singularly innervated, as previously described [33], demonstrating that multiple innervation did not influence the stability of the presynaptic terminals. We found no evidence of fiber branching between multiple fibers in the EDL like previously shown for the soleus muscle [41]. It would be interesting to examine whether a multi-syncytial reticulum could influence innervation of the *mdx* myofibers, as myofibers are normally singularly innervated. It is also unlikely that the ultra-terminal sprouting results from the absence of dystrophin within the central nervous system (CNS). Full-length dystrophin stabilizes GABAergic receptor clusters in the CNS [42]. However, impaired inhibitory innervation in adult mice would predictably increase motor nerve excitation and restriction of the motor axon branches [25]. Furthermore, the *mdx*^*4cv*^ mouse strain retains the truncated Dp116 dystrophin isoform in Schwann cells such that axon conductance is unlikely to be changed [43], and no myofiber abnormalities have been observed in transgenic *mdx* mice expressing full-length dystrophin only in striated muscle [44]. While the profound changes to innervation are an integral part of the dystrophic pathophysiology in *mdx* mice, we found no change in the total number of synapses that would be consistent with the formation of new, innervated myofibers.

Myofiber branching ensures that the developing myotubes are innervated irrespective of the site of injury. Considering the increased muscle in our study resulted almost entirely from myofiber branching, it is likely that myofiber branching helps to maintain the peak force capacity of *mdx* muscles [13]. While there are clear benefits of myofiber branching, the branch points in *mdx* mice are primary sites of calcium mishandling and contraction-induced injury [33,45-47]. Thus, it would be preferable to reverse muscle loss with the formation of entirely new, innervated myofibers. Multiple studies have suggested that prospective cellular therapies for DMD can form new fibers in *mdx* muscles. For instance, direct administration of fibroblasts genetically modified to form myofibers can form multiple dystrophin positive myotubes in *mdx*^*4cv*^ muscles, although it was not determined whether these were either innervated or attached to pre-existing myofibers [48]. Delivery of isolated satellite cells contributed to the regeneration of a myofiber that only expressed the donor GFP and not the recipient DsRed [49]. Transplantation of myogenic progenitors derived from induced pluripotent stem cells can also contribute to innervated fibers within the recipient [50]. We also found that myofiber transplantation increased the number of myofibers in transverse sections of aged (28-32 months) tibialis anterior muscles [51]. However, none of these studies demonstrated that the donor cells formed a whole innervated myofiber without the contribution from the endogenous host cells. Our current study demonstrates that the formation of new, innervated myofibers is not a natural process of muscle regeneration in the *mdx*^*4cv*^ mice. However, it remains important to examine whether various experimental manipulations utilizing diverse prospective cellular therapies and delivery techniques can form new, innervated muscle fibers, which could be important for reversing the loss of muscle mass in older DMD patients.

## Conclusions

Muscle hypertrophy is a transitory feature of some muscles in DMD and is a prevalent phenotype of the *mdx* mouse model of DMD. Analysis of muscle histopathology suggests the formation of new myofibers contributes to the hypertrophy. New myofibers require innervation to survive. However, it is not clear whether the regenerative response to muscle necrosis in *mdx* mice leads to the formation of new, innervated myofibers. Here, we demonstrate that the new muscle receives innervation by branching from the existing myofibers, rather than directly from the motoneuron. Furthermore, the propensity of myofibers to branch increased once the *mdx* mouse had reached maturity. These results have important implications for muscle replacement strategies for muscular dystrophy and sarcopenia.

## Abbreviations

CFP: Cyan fluorescence protein; DMD: Duchenne muscular dystrophy; EDL: Extensor digitorum longus; GAP 43: Growth associated protein 43; PBS: Phosphate buffered saline.

## Competing interests

The authors declare they have no competing interests.

## Authors’ contributions

RMF contributed to experimental design, performed experiments, and helped draft the manuscript. JKH imaged the 5-month *mdx* innervation and edited the manuscript. JSC contributed resources and edited the manuscript. GBB conceived the project, contributed to all experimental designs, performed experiments, and helped draft and edit the manuscript. All authors read and approved the final manuscript.

## Supplementary Material

Additional file 1: Figure S1 Detailed examination of the small AChR patches revealed them to be either **(A)** activated CD4 T-helper lymphocytes expressing interferon-γ, **(B)** CD8 cytotoxic T lymphocytes, or most prominently **(C)** macrophages. The CFP is pseudo-colored in yellow in **A-C** to allow the presentation of multiple fluorophores. We found no direct association between the immune cells and the synapse. Scale bars for **A-C** = 30 μm. Click here for file

Additional file 2: Figure S2 Synapse numbers in the third compartment of the EDL in wild-type mice does not change between 4 weeks and 12 weeks of age (n = 4). Click here for file

Additional file 3: Figure S3 Shown are **(A)** a singularly innervated myofiber and **(B)** an example of a rare myofiber with multiple synapses. Click here for file
