# Supplementary figures and images for: Myofiber branching rather than myofiber hyperplasia contributes to muscle hypertrophy in mdx mice
Source: Skelet Muscle. 2014 May 23;4:10. doi: 10.1186/2044-5040-4-10 (PMC4047439; doi:10.1186/2044-5040-4-10)

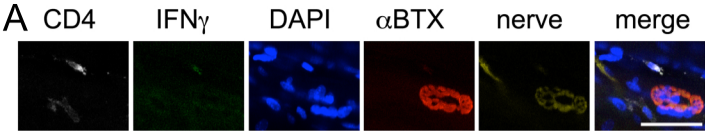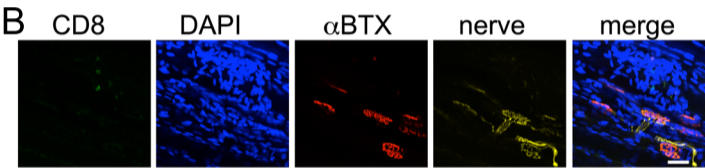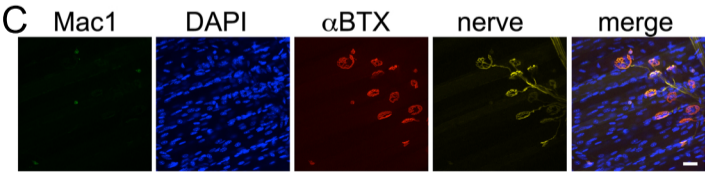

Supplement: Additional file 1: Figure S1 — Detailed examination of the small AChR patches revealed them to be either (A) activated CD4 T-helper lymphocytes expressing interferon-γ, (B) CD8 cytotoxic T lymphocytes, or most prominently (C) macrophages. The CFP is pseudo-colored in yellow in A-C to allow the presentation of multiple fluorophores. We found no direct association between the immune cells and the synapse. Scale bars for A-C = 30 μm. [file 2044-5040-4-10-S1.pdf]

**Synapse number**

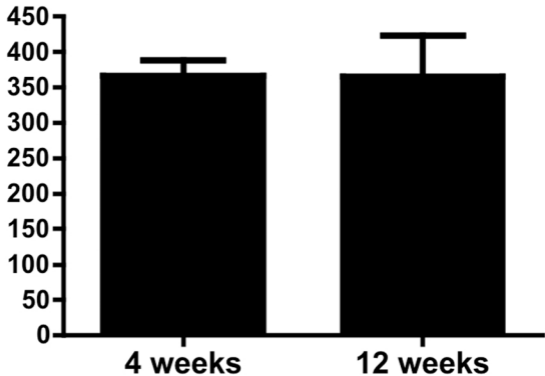

Supplement: Additional file 2: Figure S2 — Synapse numbers in the third compartment of the EDL in wild-type mice does not change between 4 weeks and 12 weeks of age (n = 4). [file 2044-5040-4-10-S2.pdf]

A

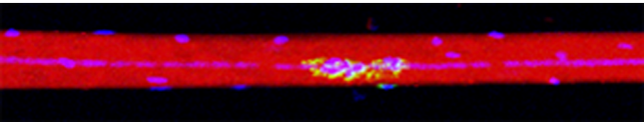

B

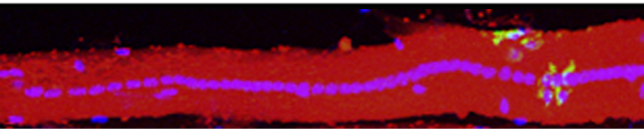

Supplement: Additional file 3: Figure S3 — Shown are (A) a singularly innervated myofiber and (B) an example of a rare myofiber with multiple synapses. [file 2044-5040-4-10-S3.pdf]
